# Single phase computed tomography is equivalent to dual phase method for localizing hyperfunctioning parathyroid glands in patients with primary hyperparathyroidism: a retrospective review

**DOI:** 10.7717/peerj.3586

**Published:** 2017-08-15

**Authors:** Fanny Morón, Alfred Delumpa, Justin Chetta, Danielle Guffey, David Dunaway

**Affiliations:** 1 Radiology, Baylor College of Medicine, Houston, TX, United States of America; 2 Dan L Duncan Institute for Clinical and Translational Research, Baylor College of Medicine, Houston, TX, United States of America

**Keywords:** Parathyroid adenoma, CT, 4DCT, Hyperparathryoidism

## Abstract

**Objective:**

This study aims to compare the sensitivity of dual phase (non-contrast and arterial) versus single phase (arterial) CT for detection of hyper-functioning parathyroid glands in patients with primary hyperparathyroidism.

**Methods:**

The CT scans of thirty-two patients who have biochemical evidence of primary hyperparathyroidism, pathologically proven parathyroid adenomas, and pre-operative multiphase parathyroid imaging were evaluated retrospectively in order to compare the adequacy of single phase vs. dual phase CT scans for the detection of parathyroid adenomas.

**Results:**

The parathyroid adenomas were localized in 83% of cases on single arterial phase CT and 80% of cases on dual phase CT. The specificity for localization of parathyroid tumor was 96% for single phase CT and 97% for dual phase CT. The results were not significantly different (*p* = 0.695). These results are similar to those found in the literature for multiphase CT of 55–94%.

**Conclusions:**

Our study supports the use of a single arterial phase CT for the detection of hyperfunctioning parathyroid adenomas. Advances in knowledge: a single arterial phase CT has similar sensitivity for localizing parathyroid adenomas as dual phase CT and significantly reduces radiation dose to the patient.

## Introduction

Primary hyperparathyroidism occurs in 0.2% to 0.5% of the general population with approximately 100,000 new reported cases per year in the United States and estimated annual incidence of 27.7 per 100 person years (Liebert, 1991; Wermers et al., 1997). Approximately 75–85% of primary hyperparathyroidism is caused by hormone overproduction by a single hyperfunctioning gland (Fraser, 2009; Ghandur-Mnaymneh & Kimura, 1984). Surgical excision of the abnormal gland is the definitive treatment (Fraser, 2009; Suliburk & Perrier, 2007).

Preoperative imaging for the purpose of localization of the hyperfunctioning gland may allow the surgeon to perform minimally invasive directed parathyroidectomy if the culprit gland is identified. Minimally invasive technique (MIT) is associated with decreased morbidity relative to four-gland exploration. This technique may be performed under local anesthesia which can be performed in candidates who may not be sutitable for general anesthesia and can be used to prevent the need for overnight hospitalization (Suliburk & Perrier, 2007).

Traditional preoperative localization uses ultrasound and sestamibi scans, which have reported sensitivities for abnormal parathyroid localization up to 74% and 58% respectively (Tublin et al., 2009). More recently, dynamic multiphase computed tomography (4DCT) using 4 phases (non-contrast and post-contrast arterial, early and delayed venous phases) shows higher sensitivities of 55–87% (Hunter et al., 2012; Rodgers et al., 2006; Starker et al., 2011). Some authors suggest that two or three phase scans may yield similar results (Hunter et al., 2014; Hunter et al., 2012; Kelly, Hamberg & Hunter, 2014).

In 4DCT, the arterial phase best discriminates between the density of hypervascular parathyroid adenomas and lymph nodes when compared to other phases (Beland et al., 2011; Raghavan et al., 2014). A 2014 study by Raghavan et al. supports single arterial phase computed tomography (CT) for routine localization of parathyroid disease. Our study aims to compare the sensitivity and accuracy of dual phase (non-contrast and arterial) versus single phase (arterial) CT for the localization of hyperfunctioning glands in patients with primary hyperparathyroidism. If localization rates are similar in the two groups, then a single phase CT would offer the same clinical benefit thereby reducing patient radiation dose and possibly increasing efficiency within the radiology department.

## Materials and Methods

Our institutional review board approved this study (H-35723). Data was collected retrospectively and de-identified in compliance with Health Insurance Portability and Accountability Act regulations. No consent was required or performed as per our internal IRB approval.

### Patient selection

Between June 2012 and October 2014, 78 patients with primary hyperparathyroidism underwent parathyroidectomy. Of these, 32 patients met the following inclusion criteria for our study. This included preoperative laboratory data consistent with primary hyperparathyroidism (elevated serum calcium and serum parathyroid hormone), preoperative dual phase neck CT performed for localization of abnormal parathyroid gland(s), and parathyroid surgical resection. The most common reason for exclusion was due to the lack of preoperative CT. Pre-operative CT was done only if sestamibi and ultrasound did not have concordant results in localizing the potential hyperfunctioning gland.

### CT technique

Patients were imaged on a 64-section multidetector CT scanner. A few patients were imaged on a 16-section multidetector CT scanner. An unenhanced phase was first obtained from the angle of the mandible to 2 cm below the carina. Then 100 mL of iodinated contrast material (Omnipaque 350) was injected at 4 mL/second via a pressure injector 25 s before an arterial phase was obtained. The same field of view was used for the arterial phase. Imaging parameters were set at 120 kVp, 150–600 mA range, gantry rotation time of 0.4 s, pitch of 1, detector configuration section width of 0.625 mm, total beam width of 40 mm, and table translational speed of 67 mm/second. Arterial phase images were acquired with a 0.625 mm slice thickness, 400 HU window width, and 50 HU window center. Axial, sagittal, and coronal images with 2.5 mm slice thickness were also reconstructed. Arterial phase maximum intensity projection (MIP) images were reconstructed with a 1.25 mm slice thickness and a 0.625 mm spacing. Images were evaluated on a Fuji Synapse PACS.

### Image analysis

Two board-certified neuroradiologists (F.M., D.D) independently reviewed each CT examination in two sessions separated by at least one week remote to the date of the operation. The readers were blinded to clinical information including surgical and pathology findings except for the diagnosis of primary hyperparathyroidism. In the first session, only the arterial phase was utilized for interpretation. The studies were again randomized and reinterpreted in a second session using both the unenhanced and arterial phases.

**Figure 1:**
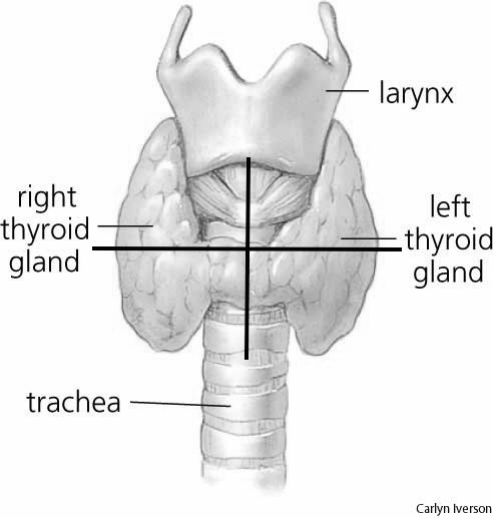
Four quadrants system used to determine localization of parathyroid gland. No overly descended glands were found on this group of patients. Photo published with permission from Carolyn Iverson.

Abnormal lesions in characteristic locations predicted by parathyroid embryology were considered possible abnormal parathyroid glands despite other imaging characteristics. Characteristic locations are usually in the midline neck, between the carotid spaces, and anterior or anterolateral to the spine. In the craniocaudal dimension, most are found between the hyoid bone and carina (Randall et al., 2009). During each interpretation if an abnormal parathyroid gland was identified, the reader was tasked with indicating the quadrant (right upper quadrant, left upper quadrant, right lower quadrant, left lower quadrant, or ectopic location of the abnormal gland) on a worksheet. Sagittal midline and transverse planes through the mid-thyroid gland defined the four quadrants (Fig. 1). An abnormal gland in an unexpected location of a normal parathyroid gland location was considered ectopic.

### Surgical technique

The surgeon used MIT when imaging localized a suspicious hyperfunctioning gland. This technique entailed limited unilateral neck dissection with resection of the lesion which was concerning by imaging. In those patients without a suspicious gland by imaging, a lengthier neck exploration was performed which included examination of all parathyroid glands.

### Pathology/biochemical analysis

All patients had biochemical data suggestive of primary hyperparathyroidism. All patients had surgically resected abnormal parathyroid glands. The surgeon reported localization of the lesions to the four quadrants or ectopic (Fig. 1). Intraoperative PTH was collected in order to assure immediate biochemical result. The pathological diagnosis was confirmed histologically. Calcium was checked following the surgical procedure to assess for an enduring result.

### Statistical analysis

Accuracy, sensitivities, specificities, and positive predictive value of localization with exact 95% confidence intervals (CI) were calculated for each reader and each session on a per quadrant basis (four quadrants and ectopic). The gold standard for diagnosis was operative reports in conjunction with histology and biochemical data. These measures were calculated and compared using generalized estimating equations (GEE) with a logit link function and exchangeable correlation matrix. The GEE model accounted for the correlation of multiple lesions within a patient and multiple readers. The estimation of sensitivity and specificity is conditional on the operative results while the estimation of positive predictive value is conditional on the imaging results. Inter-reader agreement was assessed with a Kappa statistic. All statistical analyses were performed using Stata v 12.1 (StataCorp, College Station, TX, USA).

## Results

A total of 32 patients (41% of all patients who underwent parathyroidectomy) were identified during the study period who met inclusion criteria. The patients ages ranged from 19 to 79 years old (median 59 years old), the majority of whom (97%) were women. Of all included patients, three had a prior neck surgery. None of the patient’s had a known or clinically suspected genetic proclivity for hyperparathyroidism.

The preoperative serum calcium ranged from 9.6–13.2 mg/dL (normal 8–10 mg/dL) and serum intact parathyroid hormone (iPTH) ranged from 64–789 mg/dL (normal 10–55 mg/dL). Intraoperative PTH was noted to fall in 94% of patients following resection as defined by the Miami criteria. Ninety-one percent had a normal calcium level result at a single time-variable point following surgery. The remaining 9% were lost to follow-up. No patients presented with persistent or recurrent disease.

A preoperative sestamibi SPECT/CT was performed in 88% of patients and 46% of scans localized a hyperfunctioning gland. Also, a preoperative neck ultrasound was performed in 81% of patients and 38% of scans localized a hyperfunctioning gland. Half of all included patients underwent MIT excision and the other half underwent exploratory excision. Patient demographics as well as pre-operative laboratory results are summarized in Table 1.

**Table 1:** Demographics and laboratory results.

	*N*	Percentage/Range
Sex (M)	31	97%
Median Age (years)	59	19–79
Prior neck surgery	3	9%
Median pre-op serum Ca (mg/dL)	11	9.6–13.2
Median pre-op serum PTH (mg/dL)	191	64–789
Pre-op SPECT/CT	28	88%
Surgical Procedure (Minimally Invasive/Explorative)	16/16	50%/50%

Of the included patients, 75% had single gland disease and 25% had multi-gland disease, defined as more than one hyperfunctioning gland. The single gland tumors weighed from 90–21,000 mg (median 600 mg) while multi-gland tumors weighed 10–1,000 mg (median 275 mg). Abnormal parathyroid gland characteristics are summarized in Table 2.

**Table 2:** Abnormal parathyroid gland characteristics.

	*N*	Percentage/Range
Number of patients with single gland disease	24	75%
Number of patients with multiple gland disease	8	25%
Median tumor weight		
Single gland disease	600 mg	90–21,000 mg
Multiple gland disease	275 mg	10–10,000 mg

The tumors were localized to quadrant or ectopic location in 83% of cases on single arterial phase CT and 80% of cases on dual phase CT. The results were not significantly different (*p* = 0.695). The specificity for localization of parathyroid tumor was 96% for single phase CT and 97% for dual phase CT. The positive predictive value for abnormal gland localization was 88% (95% CI [77–94]) and 89% (95% CI [78–95]) for single and dual phase scans respectively. Overall accuracy for correctly localizing a hyperfunctioning gland for single and dual phase scans is 93% (95: CI [86–96]) and 92% (95% CI [86–96]) respectively. Results are summarized in Table 3.

**Table 3:** Localization to a quadrant.

	Accuracy	Sensitivity	Specificity	Positive predictive value
Single phase	93% (86–96)	83 (73–90)	96 (93–98)	88 (77–94)
Dual phase	92% (86–96)	80 (70–88)	97 (92–99)	89 (78–95)

The overall inter-reader agreement was calculated at each location. The single phase CT had an overall kappa score of 0.705 (CI [0.650–0.742]) and dual phase CT had an overall kappa score of 0.595 (CI [0.398–0.698]). These Kappa scores indicate substantial agreement.

## Discussion

Multiple authors advocate multiphase CT scans for the localization of parathyroid adenomas (Bahl et al., 2015; Griffith et al., 2015; Hunter et al., 2012; Raghavan et al., 2014; Rodgers et al., 2006; Starker et al., 2011). These protocols utilize up to four separate phases. The sensitivity of localization of parathyroid adenomas in the literature has been reported to be as high as 55–94%. Our results demonstrate sensitivities for localization of 81.4% (95% CI [74.8–86.9]) when using a single arterial phase protocol.

Our study also demonstrates a Cohen’s kappa score of 0.705 (0.650–0.742) on single-phase studies, which indicates a substantial inter-observer agreement. The high concordance between readers is evidence of generalizable results. The difference in concordance may be due in part to the lack of a strict imaging criteria aside from location. Abnormal lesions in characteristic locations by embryology were considered potential parathyroid glands despite other imaging characteristics.

Hyperenhancement of hyperfunctioing parathyroid glands is well-described and a good diagnostic clue (Randall et al., 2009). However, atypical hypoenhancing adenomas have more recently been described; thus, strict criteria based on lesion density was not utilized (Bahl et al., 2015). Some cases remain non-diagnostic when using arterial hyperenhancement alone. Non-arterial and delayed phases in parathyroid CT protocols have traditionally been used in order to solve these scans. However, challenges which would be resolved with additional phases may not be significant contributors to the number of false negative or non-diagnostic scans.

Many of the non-diagnostic scans reported in prior studies resulted from characteristics unrelated to enhancement (Hoang et al., 2014). These include glands which are obscured by streak artifact (related to IV contrast bolus or patient’s shoulders), cystic changes, intralesional hemorrhage, and altered vascular supply from prior surgery, thyroiditis or goiter (Hoang et al., 2014). The same characteristics resulted in many of the false negatives in our study as well and would not be remedied with the use of additional scans.

**Figure 2:**
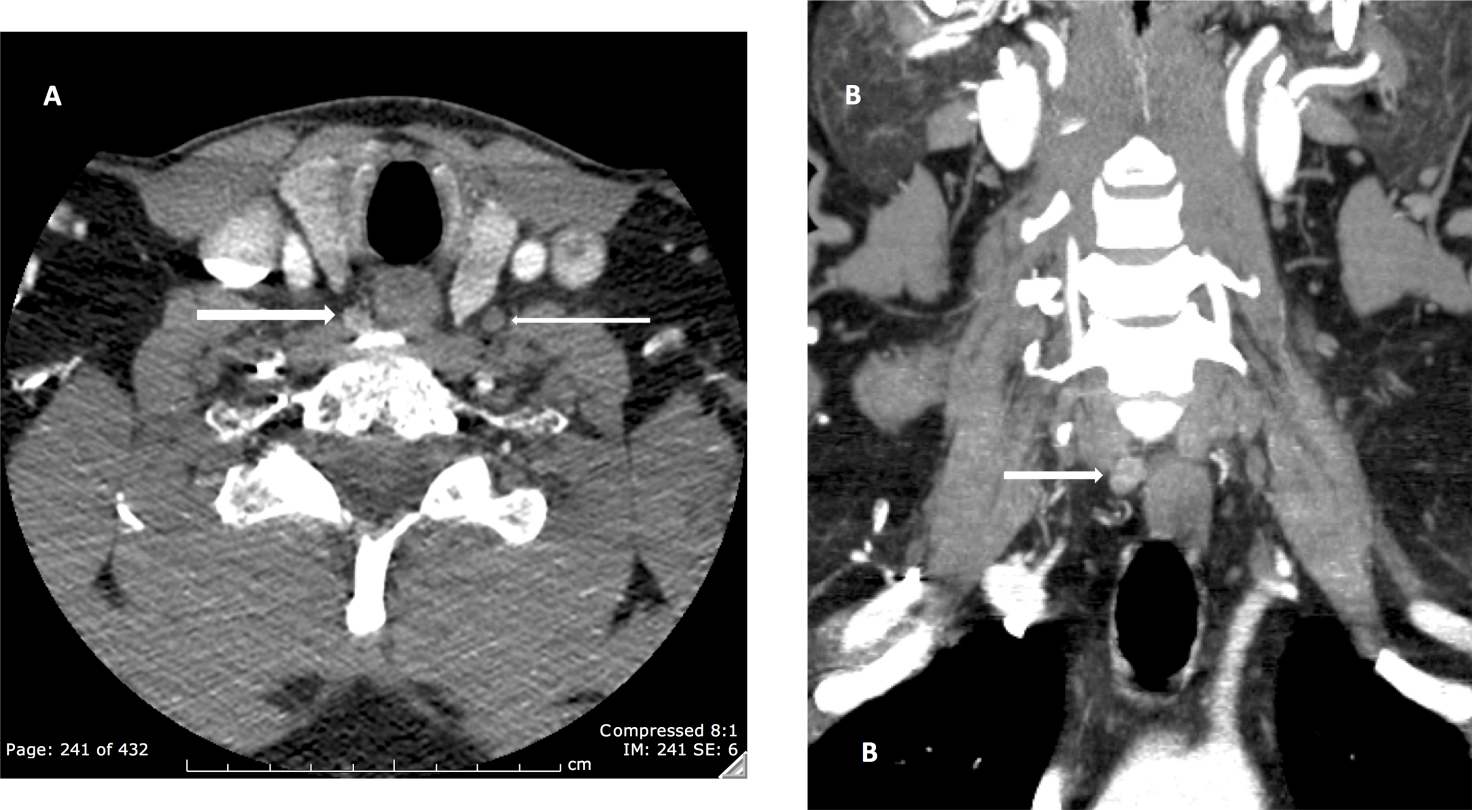
Right upper quadrant parathyroid adenoma. (A and B) Axial and coronal images, the adenoma enhances to 140 HU (thick arrow) while the lymph node enhances to 45 HU (thin arrow).

Another common use for additional phases is for discriminating a regional lymph node from a hyperfunctioning parathyroid gland. The radiologists in our study did not encounter difficulty separating parathyroid lesions from regional lymph nodes given that hyperfunctioning glands enhance to a value 40–60 Hounsfield units (HU) more on arterial phase than what would be expected for an enhancing lymph node based on multiple prior enhancement versus time models (Beland et al., 2011; Gafton et al., 2012; Raghavan et al., 2014) (Fig. 2). A parathyroid adenoma would also be expected to be lobulated in morphology possibly with the presence of a polar vessel (an enlarged feeding artery or draining vein) (Bahl et al., 2014; Hoang et al., 2014).

**Figure 3:**
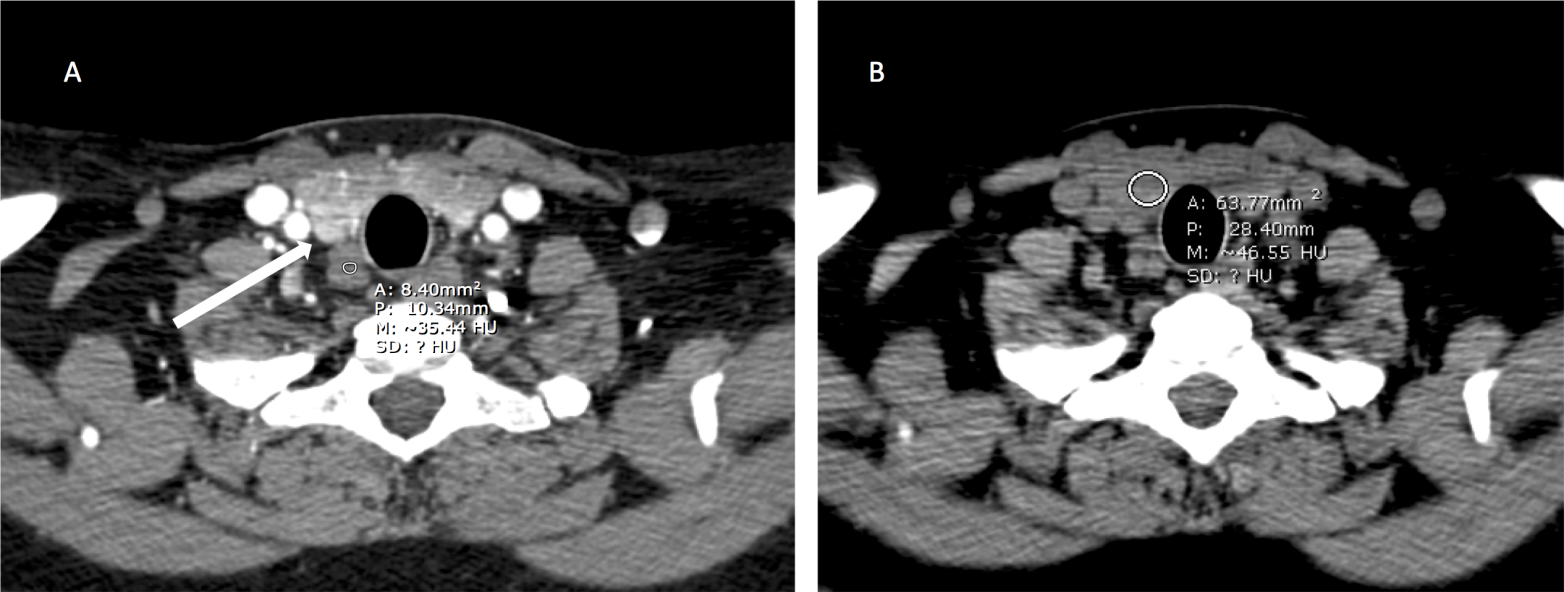
Right lower quadrant parathyroid adenoma. (A) Axial arterial phase, the adenoma enhances to 160 HU(thick arrow),while the lymph node enhances close to 35 HU (elliptical ROI). (B)The non-contrast image of a hypo-attenuating thyroid does not contribute to the interpretation (elliptical ROI close to 35 HU). (C–D) Post contrast arterial coronal and sagittal images help localize the adenoma.

There are examples where an additional phase may provide utility. An intrinsically hyper-attenuated exophytic thyroid nodule may be difficult to differentiate from an enhancing parathyroid adenoma without an unenhanced scan. This was not a significant contributor to the number of false negative scans in our study. There are also instances in which the thyroid gland is hypo-attenuating, usually from prior thyroiditis, and a non-contrast scan would not add information to the interpretation of the study (Fig. 3).

Limiting the number of scans in a protocol is of particular importance because it is an effective way to lower radiation dosage (Costello et al., 2013). Our study reduces radiation exposure by up to one-half while obtaining the same diagnostic information. This is especially important for younger patients whose lifetime attributable risk is significantly increased by the added radiation exposure (National Cancer Institute, 2009).

There are some limitations of this study. The study is retrospective and the sample size is small. Only patients with primary hyperparathyroidism were included, thus the results are not generalizable for other patient populations. In addition, the single and dual phase studies were read by the same radiologists, which may result in memory bias. This bias was mitigated by reading the studies at least one week apart and de-identifying the patient information.

## Conclusion

In conclusion, our study supports the use of a single arterial phase CT for the localization of hyper-functioning parathyroid glands. Our study found similar sensitivity and specificity of a single phase CT when compared to a dual phase CT in this specific patient population. A single-phase study may result in a reduction of radiation exposure by up to one-half, making the single phase scan a beneficial alternative to a dual phase scan. Further larger-scale studies are needed to confirm our findings.

##  Supplemental Information

10.7717/peerj.3586/supp-1 Data S1 Raw data

## Additional Information and Declarations

### Data Availability

The following information was supplied regarding data availability: The raw data has been supplied as Data S1.
