# Supplementary material for: Single phase computed tomography is equivalent to dual phase method for localizing hyperfunctioning parathyroid glands in patients with primary hyperparathyroidism: a retrospective review
Source: PeerJ. 2017 Aug 15;5:e3586. doi: 10.7717/peerj.3586 (PMC5562135; doi:10.7717/peerj.3586)
Supplement: Data S1 [file peerj-05-3586-s001.docx]

Moron – parathyroid adenoma CT data analysis summary 12/8/14

Methods:

CT and OP results were compared by each Reader/Phase combination. Accuracy, sensitivity and specificity were calculated with an exact 95% confidence interval. This comparison overall was compared in a similar manner but accounted for patients having multiple lesions and multiple readers using generalized estimating equations.

Kappa statistics, with 95% confidence intervals, were calculated for comparing phases as well as for comparing readers.

Results:

Table 1: Comparing CT and OP by location and lateralization for each reader/Phase

| Reader 1 Phase 1 | | |  |
| --- | --- | --- | --- |
|  | Accuracy | Sensitivity | Specificity |
| RU | 100 | 100 (73.5, 100) | 100 (83.2, 100) |
| RL | 90.63 | 100 (71.5, 100) | 85.7 (63.7, 97) |
| LU | 90.63 | 57.1 (18.4, 90.1) | 100 (86.3, 100) |
| LL | 93.75 | 81.8 (48.2, 97.7) | 100 (83.9, 100) |
| ectopic | 84.38 | 50 (1.26, 98.7) | 86.7 (69.3, 96.2) |
| Left | 87.5 | 76.5 (50.1, 93.2) | 100 (78.2, 100) |
| Right | 90.63 | 100 (85.2, 100) | 66.7 (29.9, 92.5) |
| Reader 1 Phase 2 | | |  |
|  | Accuracy | Sensitivity | Specificity |
| RU | 96.8 | 100 (73.5, 100) | 95 (75.1, 99.9) |
| RL | 93.75 | 100 (71.5, 100) | 90.5 (69.6, 98.8) |
| LU | 90.63 | 57.1 (18.4, 90.1) | 100 (86.3, 100) |
| LL | 90.63 | 72.7 (39, 94) | 100 (83.9, 100) |
| ectopic | 90.63 | 50 (1.26, 98.7) | 93.3 (77.9, 99.2) |
| Left | 84.38 | 70.6 (44, 89.7) | 100 (78.2, 100) |
| Right | 90.63 | 100 (85.2, 100) | 66.7 (29.9, 92.5) |
| Reader 2 Phase 1 | | |  |
|  | Accuracy | Sensitivity | Specificity |
| RU | 96.88 | 91.7 (61.5, 99.8) | 100 (83.2, 100) |
| RL | 93.75 | 90.9 (58.7, 99.8) | 95.2 (76.2, 99.9) |
| LU | 93.75 | 71.4 (29, 96.3) | 100 (86.3, 100) |
| LL | 84.38 | 63.6 (30.8, 89.1) | 95.2 (76.2, 99.9) |
| ectopic | 96.88 | 50 (1.26, 98.7) | 100 (88.4, 100) |
| Left | 81.25 | 70.6 (44, 89.7) | 93.3 (68.1, 99.8) |
| Right | 90.63 | 91.3 (72, 98.9) | 88.9 (51.8, 99.7) |
| Reader 2 Phase 2 | | |  |
|  | Accuracy | Sensitivity | Specificity |
| RU | 93.75 | 83.3 (51.6, 97.9) | 100 (83.2, 100) |
| RL | 90.63 | 90.9 (58.7, 99.8) | 90.5 (69.6, 98.8) |
| LU | 93.75 | 71.4 (29, 96.3) | 100 (86.3, 100) |
| LL | 84.38 | 63.6 (30.8, 89.1) | 95.2 (76.2, 99.9) |
| ectopic | 96.88 | 50 (1.26, 98.7) | 100 (88.4, 100) |
| Left | 81.25 | 70.6 (44, 89.7) | 93.3 (68.1, 99.8) |
| Right | 87.5 | 87 (66.4, 97.2) | 88.9 (51.8, 99.7) |

Table 2: Comparing CT to OP overall (for all locations/lateralization)

| By lat | Accuracy | Sensitivity | Specificity |
| --- | --- | --- | --- |
| All | 86.72 | 85 (78.5, 90.1) | 98.58 (81.7, 94.9) |
| R1P1 | 89.06 | 90 (76.3, 97.2) | 87.5 (67.6, 97.3) |
| R1P2 | 87.5 | 87.5 (73.2, 95.8) | 87.5 (67.6, 97.3) |
| R2P1 | 85.94 | 82.5 (67.2, 92.7) | 91.67 (73, 99) |
| R2P2 | 84.38 | 80 (64.4, 90.9) | 91.67 (73, 99) |
| Phase 1 | 87.5 (75.14, 94.19) | 86.25 (76.7, 92.9) | 89.58 (77.3, 96.5) |
| Phase 2 | 85.94 (74.51, 92.74) | 83.75 (73.8, 91.1) | 89.58 (77.3, 96.5) |
|  |  |  |  |
| By loc | Accuracy | Sensitivity | Specificity |
| All | 92.34 | 81.4 (74.8, 86.9) | 96.37 (94.2, 97.9) |
| R1P1 | 91.88 | 86.05 (72.1, 94.7) | 94.02 (88.1, 97.6) |
| R1P2 | 92.5 | 83.7 (69.3, 93.2) | 95.73 (90.3, 98.6) |
| R2P1 | 93.13 | 79.07 (64, 90) | 98.29 (94, 99.8) |
| R2P2 | 91.88 | 76.74 (61.4, 88.2) | 97.44 (92.7, 99.5) |
| Phase 1 | 92.50 (85.62, 96.23) | 82.56 (72.9, 89.9) | 96.15 (92.8, 98.2) |
| Phase 2 | 92.19 (86.37, 95.65) | 80.23 (70.2, 88) | 96.58 (92.4, 98.5) |

R1P1=Reader1 Phase1

The difference in accuracy between phase 2 and phase 1 is not statistically significant (risk difference: -0.04; 95% CI=-0.56, 0.47; p=0.866) for localization. The difference in accuracy between phase 2 and phase 1 is not statistically significant (risk difference: -0.14; 95% CI=-0.46, 0.37; p=0.597) for lateralization.

Table 3: Kappa Statistics with 95% Confidence Intervals

|  | Comparing Phases | | Comparing Readers | |
| --- | --- | --- | --- | --- |
|  | Reader 1 | Reader 2 | Phase 1 | Phase 2 |
| RU | 0.934 (0.808, 1) | 0.929 (0.793, 1) | 0.932 (0.802, 1) | 0.798 (0.586, 1) |
| RL | 0.936 (0.813, 1) | 0.932 (0.802, 1) | 0.805 (0.599,1) | 0.803 (0.592, 1) |
| LU | 1 (1, 1) | 0.763 (0.449, 1) | 0.871 (0.624, 1) | 0.613 (0.216, ) |
| LL | 0.92 (0.766, 1) | 0.833 (0.611, 1) | 0.76 (0.504, 1) | 0.667 (0.366, 0.967) |
| ectopic | 0.717 (0.352, 1) | 1 (1, 1) | 0.297 (-0.162, 0.755) | 0.475 (-0.124, 1) |
| Left | 0.934 (0.808, 1) | 0.741 (0.504, 0.978) | 0.870 (0.697, 1) | 0.672 (0.409, 0.935) |
| Right | 1 (1, 1) | 0.929 (0.793, 1) | 0.674 (0.391, 0.956) | 0.612 (0.324, 0.9) |
